# Evaluating *in vivo* approaches for studying the roles of thymic DCs in T cell development in mice

**DOI:** 10.3389/fimmu.2024.1451974

**Published:** 2024-08-06

**Authors:** Yi Wang, Mark M. W. Chong

**Affiliations:** ^1^ RNA and T cell Biology, St Vincent’s Institute of Medical Research, Fitzroy, VIC, Australia; ^2^ Department of Medicine, University of Melbourne, Parkville, VIC, Australia

**Keywords:** thymus, T cell development, central tolerance, dendritic cells, mouse models

## Abstract

T cells express an enormous repertoire of T cell receptors, enabling them to recognize any potential antigen. This large repertoire undergoes stringent selections in the thymus, where receptors that react to self- or non-danger-associated- antigens are purged. We know that thymic tolerance depends on signals and antigens presented by the thymic antigen presenting cells, but we still do not understand precisely how many of these cells actually contribute to tolerance. This is especially true for thymic dendritic cells (DC), which are composed of diverse subpopulations that are derived from different progenitors. Although the importance of thymic DCs has long been known, the functions of specific DC subsets have been difficult to untangle. There remains insufficient systematic characterization of the ontogeny and phenotype of thymic APCs in general. As a result, validated experimental models for studying thymic DCs are limited. Recent technological advancement, such as multi-omics analyses, has enabled new insights into thymic DC biology. These recent findings indicate a need to re-evaluate the current tools used to study the function of these cells within the thymus. This review will discuss how thymic DC subpopulations can be defined, the models that have been used to assess functions in the thymus, and models developed for other settings that can be potentially used for studying thymic DCs.

## Introduction

T cells undergo random somatic recombination of Variable (V), Diversity (D) and Joining (J) gene segments to assemble their T cell receptors (TCR) (1). This rearrangement theoretically allows 10^18^ possible TCRs to be generated, which must undergo strict selection checkpoints in the thymus to purge non-functional TCRs as well as those than might recognize self-antigens (2). The first checkpoint, positive selection, selects TCRs that recognize major histocompatibility complex (MHC) molecules (3). The second, negative selection, determines the self-reactivity of TCRs by displaying self-peptides on MHC molecules to thymocytes (4). Thymocytes that show strong self-reactivity are deleted or further differentiate into regulatory T cells (Treg) with immunomodulatory functions (2, 5, 6). These two checkpoints are essential for shaping the T cell repertoire. Both checkpoints require antigen presenting cells (APC) in the thymus to interact with TCRs. Therefore, they play pivotal roles in achieving the intricate balance between immunodeficiency and autoimmunity.

APCs in the thymus include the classical hematopoietic APCs, and non-classical thymic epithelial cells (TEC) (7). The role of TECs in supporting T cell development are well characterized relative to hematopoietic APCs. Three lineages of hematopoietic APCs have been identified in the thymus: dendritic cells (DC), macrophages and B cells (8–10). Of these, there is evidence demonstrating that DCs and B cells present antigens and participate in negative selection (8, 11–13). Although it remains unclear if macrophages participate in thymocyte selection, these cells can present antigens *ex vivo* (14).

From the study of transgenic mouse models that lack thymic DCs, we know that these cells play non-redundant roles in clonal deletion of self-reactive CD4 T cells and induction of Tregs (15). Despite the importance of thymic DCs, the lineage origins of these cells are not well characterized. As a result, it is difficult to manipulate individual DC subsets to untangle the functions of these subsets. More recent studies that better define DC biology in the thymus and other organs have been enabled by new technologies such as transcriptomic profiling, allowing us to utilize mouse models to manipulate these cells more accurately (**Table 1**).

**Table 1:** Summary of genetically modified mouse models for studying DC functions.

Model	Full nomenclature	Publication	Thymus assessed?	DC population impacted	Observed T cell development phenotype
CD11c-DTA	CD11c-Cre/R-DTA	(15)	Yes	Total DC	Impaired CD4 negative selection
CD11c-DTR	B6.FVB-1700016L21Rik^Tg(Itgax-HBEGF/EGFP)57Lan^/J	(16)	Yes	Total DC	Impaired development of MJ23 Treg clone
Zbtb46-DTR/zDC-DTR	B6(Cg)-*Zbtb46^tm1(HBEGF)Mnz^*/J	(17)	Yes	Total cDC	/
Batf3-KO	B6.129S(C)-*Batf3^tm1Kmm^*/J	(16)	Yes	cDC1	cDC1 augment mTEC in central tolerance
IRF8-KO	B6(Cg)-*Irf8^tm1.2Hm^*/J	(16, 18)	Yes	cDC1	cDC1s were dispensable for MJ23 Treg clone
XCR1-DTR	Xcr1^tm2(HBEGF/Venus)Ksho^	(19)	No	cDC1	/
Clec9a-DTR	Clec9a^Cre/Cre^Rosa**^DTR^**	(20)	No	cDC1	/
CD103-DTR	CD103-LoxP-floxed stop cassette + DTR x CD11c-Cre	(21)	Yes	cDC1	cDC1s were dispensable for microbiota-specific CD4 T cell development
IRF4-KO	B6.129P2-*Irf4^tm1Mak^*/J	(22–24)	No	cDC2	/
Zeb2-KO	B6;129(Cg)-*Zfhx1b^tm1.1Yhi^*	(25)	No	cDC2, pDC	/
NFIL3-C/EBP mutant mice		(26)	No	cDC2, monocytes	/
Mgl2-DTR eGFP	B6(FVB)-*Mgl2^tm1.1(HBEGF/EGFP)Aiwsk^*/J	(27)	Yes	CD301b+cDC2	Depletion of CD301b+ cDC2s impairs clonal selection
IL4Ra-KO	FVB/NJ-*Il4ra^em1Amen^*/J	(27)	Yes	CD301b+cDC2	/
CX3CR1-DTR	B6N.129P2-*Cx3cr1^tm3(Hbegf)Litt^*/J	(21)	Yes	CX3CR1+DC	CX3CR1+DCs support microbiota-specific CD4 T cell development
Notch2-cKO	CD11c^cre^ x B6.129S-*Notch2^tm3Grid^*/J	(28)	No	Notch2-dependent cDC2A	/
KLF4-cKO	CD11c^cre^ x B6.129S6-*Klf4^tm1Khk^*/Mmmh	(29)	No	KLF4-dependent cDC2B	/
BDCA2-DTR	C57BL/6-Tg(CLEC4C-HBEGF)956Cln/J	(16, 30, 31)	Yes	BDCA2+ pDC	affect innate lymphocyte development; dispensable for MJ23 Treg clone and activating iNKT2 cells
MHC II-KO	B6.129S2-*H2^dlAb1-Ea^*/J	(17)	Yes	cDC maturation	Significantly reduced mature DC
CD40-KO	B6.129P2-Cd40^tm1Kik^/J	(17)	Yes	cDC maturation	CD40-deficient DCs mature poorly
XCL1-KO		(32)	Yes	cDC1 migration	Impaired natural Treg generation
CCR2-KO	B6.129S4-*Ccr2^tm1Ifc^*/J	(33)	Yes	cDC2 migration	Impaired blood-borne-antigen-specific T cell development
CCR9-KO	B6N.129-*Ccr9^tm1Lov^*/JmfJ	(31, 34)	Yes	pDC migration	affect peripheral antigen transport and innate lymphocyte development

## Manipulating total DCs

The importance of thymic DCs was revealed by exploiting their common expression of CD11c. One of these approaches is diphtheria toxin- (DT) mediated cell knockout models (35, 36). Mice transgenic for CD11c-cre and flox-stop- diphtheria toxin α chain (DTA), which ablates all CD11c-expressing cells, have been used to study the consequence on T cell development (15). In the thymi of these mice, the majority of conventional DCs are depleted and negative selection is severely impaired in the CD4 compartment. Using the same model, another study demonstrated poor development of Treg clones (37). In the CD11c-DTR model, where DCs are depleted upon DT administration, thymic DCs were shown to support the development of a prostate antigen-specific Treg clone (16).

Although CD11c is generally agreed to be a DC-specific marker, a recent study showed that thymic macrophages also express CD11c, as well as class II major histocompatibility complex (MHC II) and SIRPα, which are canonical markers for conventional DC2s (cDC2) (14). This is despite having transcriptional profiles that are clearly distinct from DCs. This suggests that macrophages could be easily mistaken as DCs in phenotypic characterization and the approaches exploiting CD11c expression are likely also affecting macrophages. An alternative marker that is thought to be exclusive to conventional DC (cDC) is the transcription factor *Zbtb46* (38). With a Zbtb46-DTR transgene, depletion of close to 80% of thymic cDCs has been reported (17, 39).

## Defining DCs by phenotypic subsets

**Figure 1:**
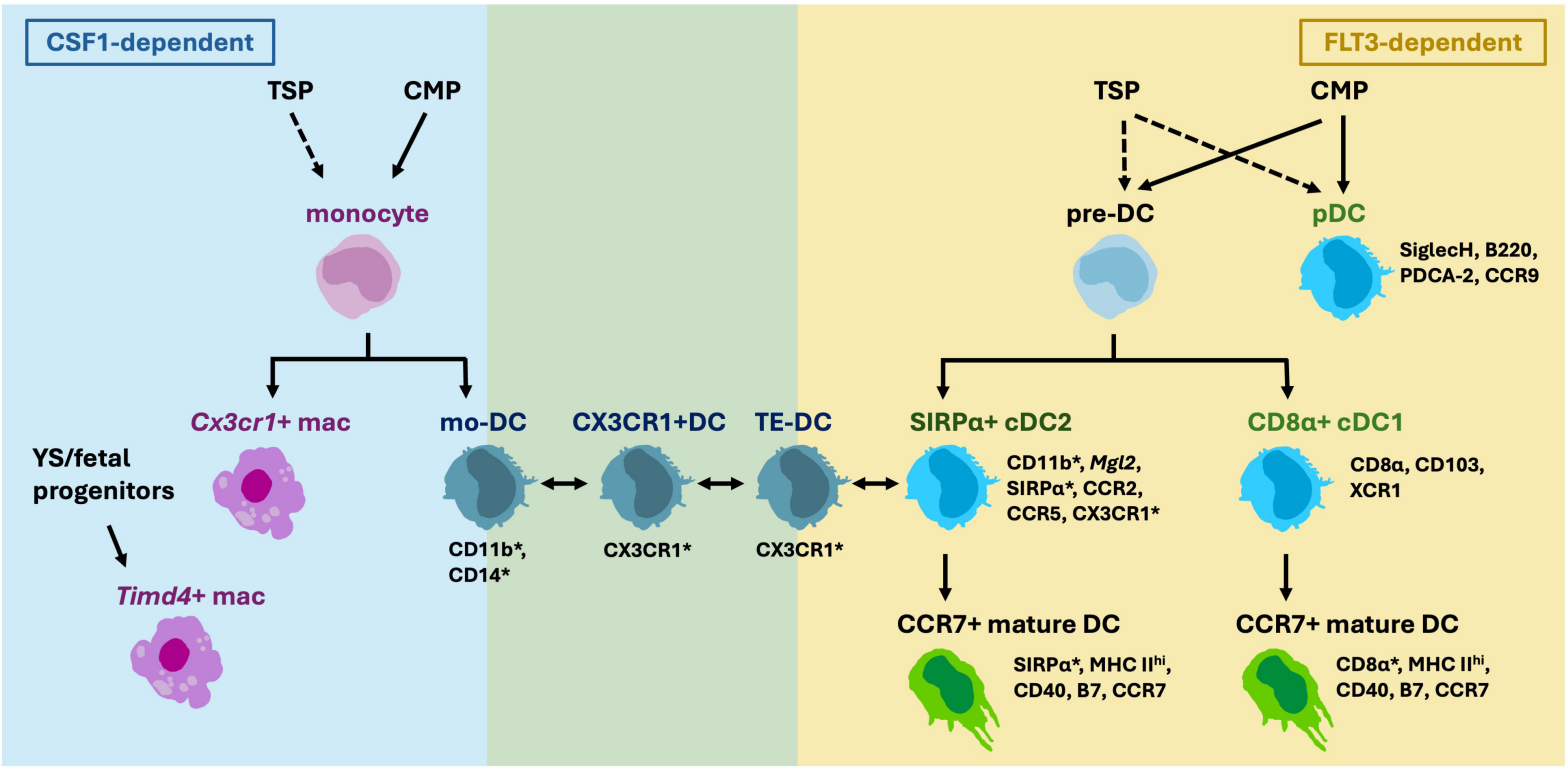
Thymic DCs are composed of various subsets that are derived from different progenitors. Thymic seeding progenitors (TSP) are suggested to give rise to thymic monocytes, pre-DC and pDCs in addition to common myeloid progenitors (CMP). Multiple CX3CR1+ cDC2-like populations have been described, and it is yet unclear whether they are derived from monocytes, or pre-DC, and whether they are CSF1- or FLT3-dependent. Overlapping Markers expressed by DC2s and macrophages (marked by asterisks), such as CD11c, MHC II, SIRPα, CD11b and CX3CR1, increase the difficulty to distinguish the cells apart.

Compared to the other hematopoietic APCs, DCs are relatively well-characterized in the thymus. However, studies on thymic DCs thus far have yet to piece together a clear picture of their composition and functions. The earliest studies identified phenotypic markers to distinguish three DC subsets in mice which are still the canonical markers currently in use: CD8α+ cDC1, SIRPα+CD11b+ cDC2 and PDCA+SiglecH+ plasmacytoid DC (pDC) (**Figure 1**) (40). cDC1s take up self-antigens expressed by medullary TECs and display them for negative selection (13). cDC2s are also known to participate in negative selection and to generate regulatory T cells (Treg) *in vitro* (18). It is unclear yet whether one subset contributes towards tolerance more than another, or whether they are functionally redundant.

## Controversies surrounding the DC2 lineage

cDCs and pDCs are derived from common DC progenitors that have lost monocyte-potential in the bone marrow (41). There are currently no agreed phenotypic markers that specifically label *bona fide* cDC2s. Commonly used markers such as SIRPα and CD11b overlap with monocyte-derived cells (14, 42–44). While the current gold standard to define macrophage and DC lineages is by their differential dependence on the growth factors CSF1 and FLT3, respectively, thymic cDC2s are often defined by only phenotypic markers that do not exclusively label cDC2s. In the thymus of Zbtb46-DTR mice, CD8α+cDC1s can be totally depleted with DT, but around 20% of “SIRPα+cDC2s” persist (17).

Recently, “monocyte-derived DCs” (mo-DC) were reported in the thymus (45). These cells are transcriptionally similar to monocytes but express cDC2 markers such as *Itgax* (CD11c), *Itgam* (CD11b) and *Sirpa*. However, whether they are derived from monocytes has not yet been determined. Another study reported the presence of “CX3CR1+DCs” in the thymus that migrate from the gut carrying microbiota antigens (21). In the gut, CX3CR1+ mononuclear phagocytes are derived from monocytes (46). An additional study reported the so called thymic “transendothelial-DCs” (TE-DC) that can present blood-borne antigens also express CX3CR1 (47). It is unknown if these three described populations overlap, or how they are different, other than their common expression of CX3CR1. It will be important to clarify whether these “CX3CR1+DCs” contain DC-progenitor- or monocyte-derived cells, and whether these cells belong to macrophage or DC lineage (14, 48) (**Figure 1**).

Moreover, heterogeneity among cDC2s has been demonstrated, at least in murine spleen. Splenic cDC2s can be subdivided into Notch2- dependent Tbet+ cDC2A and KLF4-dependent RORγt+ cDC2B subsets (28, 29, 49). No study has directly compared thymic cDC2s to these splenic subsets and thus heterogeneity of thymic cDC2s remain unclear.

## Manipulating cDC1s

The transcription factors required for murine cDC1 specification are well defined, and thus there are multiple mouse models that disrupt the development of cDC1s (50). The BATF3- and IRF8-deficient models affect cDC1s (51–53). The thymi of these mice have been characterized and a clear depletion of cDC1s can be seen (16, 18). However, IRF8 deficiency also affects splenic B cells and macrophages (54). It is yet unclear whether thymic B cells and macrophages are impacted, and thus the changes in T cell development observed in IRF8-deficient thymi cannot be solely attributed to the depletion of cDC1s. Alternatively, there are the CD103-DTR, XCR1-DTR or Clec9A-DTR models that utilize cDC1-specific promoters to drive DTR expression to deplete the population (19–21). The integrin CD103 is a common marker used to define intestinal DC1s (55). As this marker is also expressed by thymic DC1s, the conditional CD103-DTR (CD11c^cre^CD103^DTR^) model has been shown to efficiently deplete cDC1s in the thymus (21). Thymic cDC1 depletion has not been characterized in XCR1-DTR or Clec9A-DTR mice.

## Manipulating cDC2s/mo-DCs

There are currently no mouse models with specific ablation of the SIRPa+ DC population. This is likely due to the possibility that this is a mixed population of cells with a similar phenotype but of different lineages. This is also the case for DC2s in other organs, where their phenotype and lineage origins are still under debate (56).

The transcription factors IRF4 and ZEB2 are implicated in the commitment of the cDC2 lineage (22–24, 57). Due to differences in environmental cues, tissue-specific cDC2s have different dependencies on IRF4. DC-specific IRF4 knockout depletes cDC2s in the lung, but only a proportion of cDC2s in the spleen and small intestinal lamina propria (22, 24). Knockout of *Zeb2* depletes cDC2s, but also pDCs (25). To specifically deplete cDC2s, a mouse model with mutations in the *Zeb2* enhancer was developed (26). Although B cells and pDCs are not impacted, monocytes are deficient in this model. No assessment of thymic cDC2s in the aforementioned mouse models have so far been reported.

Since the transcriptional regulation of cDC2s is difficult to exploit, other approaches that employ other markers expressed by thymic DC2s in DTR models have been developed, but none achieve total depletion. The *Mgl2*-DTR model specifically targets *Mgl2*/CD301b-expressing cells (58). These were found to be 30-60% of the thymic cDC2s (30, 45). The cytokine receptor IL4R regulates the maintenance of CD301b+ cDC2s and Il4Ra-deficient mice exhibit the same degree of depletion as the CD301b-DTR mice (27). CX3CR1-DTR is also a potential tool for depleting cDC2s (21). Using this model, CX3CR1+DCs were shown to be crucial for the development of microbiota-specific CD4 T cells.

The lack of mouse models with effective cDC2 depletion highlights the need to clarify the identity of the SIRPα+ myeloid cells in the thymus. The possibility that there are cDC2s, mo-DCs and macrophages with similar phenotypes implicates that the thymic cDC2s described in previous studies could in fact be comprised of a mixture of populations. The populations of different lineages should be targeted using different approaches. It will also be beneficial to map thymic cDC2s to the well-defined subsets of splenic cDC2s, which could allow existing models such as the Notch2- and Klf4- conditional knockout to be adapted for studying their functions (28, 29).

## Manipulating pDCs

pDCs express the unique surface marker CLEC4C/BDCA2 and this has been utilized for DTR transgenic models (59). There is efficient depletion of thymic pDCs in this model and they were found to be required for the development of microbiota-specific innate lymphocytes (16, 30, 31).

## Defining DCs by activation status

Circulating DCs, or immature DCs are known to undergo maturation upon uptake of an antigen and home to lymph nodes to activate T cells (60). This maturation process refers to the activation of developmentally mature and functional DCs, and not the differentiation of uncommitted progenitors into DCs. Mature DCs upregulate MHC II as well as the costimulation molecules CD80, CD86 and CD40. Mature DC homing to the thymus was first explored in 2006, and it was found that immature DCs are preferentially recruited to the thymus compared to immunogenic LPS-induced mature DCs (61). The authors propose that preferential recruitment of immature and tolerogenic DCs is a safety checkpoint in central tolerance, since DCs that mature with danger signals can lead to undesirable deletion of danger-associated thymocytes.

Using RNA sequencing, the transcriptional changes that take place during maturation of thymic CD8a+ cDC1s were found to resemble maturation of tolerogenic DCs in the periphery (62). Another study reported that both cDC1s and cDC2s undergo maturation in the thymus, and mature cDC1s are transcriptionally very similar to cDC2s (17). Unlike peripheral DCs that become less phagocytic upon maturation, this study suggested that thymic mature DCs are as efficient as immature DCs in the uptake of antigens. Other than antigen presentation, mature DCs have also been found to promote thymic atrophy via the Jagged-Notch2 axis (63). However, it has not been fully resolved whether all DC maturation takes place in the thymus or some mature DCs are recruited (17, 63).

## Manipulating DC maturation

Thymic mature DCs are found to depend on cognate MHC II-TCR interactions with CD4 single positive (SP) thymocytes and CD40-mediated costimulation signal (17). Thymic DCs that are deficient for MHC II or CD40 mature poorly. However, CD4 single positive thymocytes are absent in MHC II-deficient mice and it is difficult to study the function of mature DCs with such great impact on the CD4 compartment (64). Further, removing MHC II or CD40 directly affects the antigen presentation capacity of all DCs in the thymus, not just mature DCs. Although there are available approaches to block DC maturation, none of these can be used to address whether mature and immature DCs are functionally distinct, or how mature DC1s are different to DC2s given their transcriptional similarity.

## Defining DCs by site of development and source of antigen

The majority of DCs originate from the bone marrow, but early thymic progenitors that enter the thymus can retain multipotency and give rise to DCs (65). This means DCs in the thymus can be either derived from a progenitor in the thymus or enter the thymus as functional DCs. This different origin of DCs can fundamentally impact their function. The unique thymic microenvironment may influence DCs to acquire specialised functions, while a migrated DC can carry antigens that are derived peripherally (66, 67). Therefore, it is desirable to know where a DC has come from when studying their function in the thymus.

The characterization of developmental origin of DCs began with parabiotic mice experiments. The cDC1s were found to be resident, while the other two subsets were found to be migratory (8, 33, 34, 68, 69). These experiments elegantly demonstrate the thymic residency of cDC1s. For the cDC2s and pDCs, such parabiotic experiments only demonstrate that cells from the periphery have migrated into the thymus. It is still possible that a proportion of thymic cDC2s and pDCs develop intrathymically. Recently, single-cell transcriptomic characterization of human thymus-seeding progenitors suggest that they give rise to pDCs and monocytes (70, 71). Further, a rearranged *Tcrd* locus can be detected in a proportion of human thymic monocytes, pDCs and cDC1s, suggesting an origin via uncommitted thymocytes (70). However, the exact origins of thymic DCs are yet to be demonstrated using *in vivo* experimental models.

## Manipulating DC by migration and antigen

DCs rely on chemokine receptors to migrate throughout lymphoid organs and tissues (72). The unique pattern of chemokine receptor expression determines where subsets migrate and serve as good markers for defining these subsets. cDC1s express XCR1 and they co-localise with XCL1-expressing mTECs in the medulla of the thymus (32). This facilitates the transfer of antigens from mTECs to cDC1s to be efficiently presented or cross-presented to thymocytes (13). cDC2s and monocyte-derived cells express CX3CR1, CCR2 and CCR5, whereas pDCs express CCR9 (21, 33, 34, 47, 73–75). These two subsets are reported to transport antigens from the periphery, including introduced exogenous antigens and microbial antigens (21, 33, 34). All these chemokine receptors can be manipulated to affect their migration into the thymus and localization within the thymus.

In XCL1-deficient mice, which lack the ligand of XCR1, the co-localization of cDC1 and mTEC is impaired, leading to defective generation of thymic Tregs (32). CCR2-deficient mice display a marked reduction in thymic SIRPα+DC2s and leads to impaired negative selection of blood-borne-antigen- and tumor-antigen-specific T cells (33, 74). No decrease in SIRPα+DC2s are seen in CCR5- or CX3CR1-deficient mice (33). When all three chemokine receptors are knocked out or blocked with inhibitors, CX3CR1+DC migration is completely ablated (21). Microbial DNA is present in the thymus as long as mice express one of the three chemokine receptors, suggesting that CX3CR1+DCs can utilize all three for migration.

In CCR9-deficient mice, significantly fewer pDCs are found in the thymus (31, 34). Consequently, peripheral antigen transport is reduced and innate-like thymocyte development is impaired, similar to BDCA2-DTR mice. The downside to manipulating chemokine receptor expression is that thymocytes rely on some of the same receptors, such as CCR9, for migration, their normal development will be affected (76).

Blocking APC migration into the thymus impacts thymic selection partly by removing the antigens they are supposed to present. Therefore, manipulating antigens in peripheral tissues is another approach for studying the functions of the migratory DCs. This can be achieved by introducing non-endogenous antigens at a specific site. For example, by coating the fluorescent protein FITC on the skin, thymus homing DCs have been shown to transport antigens from the skin (61). Otherwise, antigens like ovalbumin (OVA) can be introduced by tissue-specific expression, such as the Cmy promoter in cardiomyocytes, or the insulin promoter in pancreatic β cells (61, 77, 78). Because OVA is not an endogenous antigen, TCR transgenic OT-I CD8+ or OT-II CD4+ T cells that recognise OVA have to be introduced (3, 79). With these approaches, the migratory potential of thymic DCs into different tissues and their roles in the development or deletion of the cognate T cells can be studied.

## Final comments

Despite the known importance of thymic DCs in tolerance, DC defects have yet to be implicated in autoimmune diseases. This presents the conundrum of whether this is because they are not involved, or because there are very limited tools to study them. The biggest roadblock to studying thymic DC functions is our incomplete understanding of their ontogeny and how subsets relate to each other. Due to the unique purpose of the thymus and its specialized microenvironment, assumptions made based on DC phenotypes in other secondary lymphoid organs are often inaccurate. Single-cell transcriptomic analyses of thymic myeloid cells revealed that non-DCs also express CD11c and presented the issue of cDC2 lineage heterogeneity. The phenotype of thymocytes and all hematopoietic APCs should be carefully evaluated in models that are thought to only impact DCs, to better interpret the reported changes in T cell development. This is also important when comparing different models to tease out the function of specific subsets. To further understand the origins and developmental regulation of thymic DCs, experimental approaches such as lineage tracing models are needed. It also remains beneficial to leverage existing understanding of (peripheral) DC biology and study the thymus phenotype in models previously used for assessing DCs in other organs. Having established and validated models for manipulating DC subsets can be used to answer the question of whether these subsets are functionally redundant and how each of them contribute to tolerance under normal and autoimmune settings.

## Author contributions

YW: Conceptualization, Writing – original draft, Writing – review & editing. MC: Conceptualization, Funding acquisition, Writing – review & editing.
